# Sodium and Potassium Intake and Cardiovascular Disease in Older People: A Systematic Review

**DOI:** 10.3390/nu12113447

**Published:** 2020-11-10

**Authors:** Carla Gonçalves, Sandra Abreu

**Affiliations:** 1 CIAFEL—Research Centre in Physical Activity, Health and Leisure, Faculty of Sport, University of Porto, 4099-002 Porto, Portugal; sandramrabreu@gmail.com; 2 CITAB, Centre for the Research and Technology of Agro-Environmental and Biological Sciences, 5001-801 Vila Real, Portugal; 3 Faculty of Nutrition and Food Sciences, University of Porto, 4099-002 Porto, Portugal; 4 Faculty of Psychology, Education and Sports, Lusófona University of Porto, 4000-098 Porto, Portugal

**Keywords:** cardiovascular disease, hypertension, older people, sodium, potassium

## Abstract

This review aims to examine the relationship of sodium and potassium intake and cardiovascular disease (CVD) among older people. Methods: We performed a literature search using PubMed and Web of Science (January 2015 to July 2020) without language restriction. Observational and experimental studies that reported the relationship between sodium, potassium, or sodium-to-potassium ratio with CVD among older adults aged higher than 60 years were included. The authors independently screened all identified studies, extracted information, and assessed the quality of included studies. Risk of bias was assessed using the Risk of Bias Assessment Tool for Nonrandomized Studies (RoBANS) for observational studies and the revised Cochrane risk-of-bias tool (RoB 2 tool) for randomized trials. Results: We included 12 studies (6 prospective cohort studies, 5 cross-sectional studies, and 1 experimental study). Five of the studies reported on sodium-to-potassium ratio (*n* = 5), and the others on potassium and/or sodium intake. Cardiovascular events (e.g., stroke and heart failure) were the most reported outcome (*n* = 9). Of the 12 studies included, five observational studies had low bias risk and the randomized controlled trial was judged as uncertain risk of bias. We found inconsistent results for the effect of the reduction of sodium intake in this population for lower risk of CVD. We found that both the increase of potassium intake and the decrease of sodium-to-potassium ratio were associated with lower risk of hypertension and CVD, particularly stroke. Conclusion: The present review suggests that both higher potassium and lower sodium-to-potassium ratio are associated with lower risk of CVD.

## 1. Introduction

The world is facing a critical healthcare challenge in rising and potentially unsustainable healthcare costs, mainly due to the increasing prevalence of unhealthy lifestyles, chronic diseases, and a growing ageing population that requires more diversified care and increased societal demands [1,2]. Indeed, the number of older persons (>60 years) in world is expected to double until 2050, when it is projected to reach nearly 2.1 billion of persons; the process of population ageing will be most advanced in Europe and North America [1]. It will be essential for countries to develop and implement policies to face an ageing population with a high burden of chronic conditions, including cardiovascular diseases (CVD). Multi-morbidity will increase the demand to strengthen disease prevention and integrate service delivery around people’s needs for health and social care.

On top of health-related age frailty, ageing people have non-communicable chronic diseases such as CVD that are the main contributors to the total burden of disease and mortality in low-, middle- and high-income countries [3,4]. CVD consists of a group of heart and blood vessels disorders that include coronary heart disease (e.g. heart attack), cerebrovascular disease (e.g., stroke), and diseases of the aorta and arteries, including hypertension and peripheral vascular disease [5]. In older persons, CVD imposes a huge burden in terms of mortality, morbidity, disability, functional decline, and healthcare costs [6]. Hypertension is the most powerful preventable risk factor for death and disability from CVD [7] and also for cognitive decline and loss of autonomy later in life [8]. Hypertension prevalence and severity increase with age, and treating healthy subjects aged 75 years and older with moderate to severe hypertension reduces non-fatal strokes, cardiovascular morbidity and mortality, and the incidence of heart failure [9]. The American College of Cardiology/American Heart Association (ACC/AHA) defines for most adults ≥65 years old a blood pressure (BP) goal of <130/80 mmHg [10], and the European Society of Cardiology/European Society of Hypertension (ESC/ESH) recommends for persons between the ages of 65 and 79 years (elderly) and above 80 years (very old) a BP target of <130–139/70–79 mmHg [11].

There is strong scientific evidence that behavioral (such as unhealthy diet) and metabolic (such as hypertension) risk factors play a key role in the etiology of CVD. High dietary intakes of salt and low intake of fruits and vegetables are linked to increase CVD risk [12,13], mainly due to its impact on BP. Both a lower sodium and a higher potassium intake have been associated with lowered BP and a reduction in CVD [14,15], particularly in adults with hypertension. A recent meta-analysis of 32 randomized controlled trials found a U-shaped relationship between potassium supplementation and BP, with stronger lowering effects in participants with hypertension and at higher levels of sodium intake [16]. In this line, the World Health Organization strongly recommends a reduction to <2000 mg/day in sodium intake (5 g/day salt) [17], and an increase in dietary potassium intake of at least 3510 mg/day to reduce BP and the risk of CVD, stroke, and coronary heart disease in adults [18]. Urinary sodium-to-potassium ratio is an alternative indicator of cardiovascular risk, and the proposed ideal ratio of sodium-to-potassium intake (1:1) is achieved when sodium and potassium intake are within WHO recommended values [18]. A recent meta-analysis of prospective and retrospective observational studies reported that higher sodium intake and higher sodium-to-potassium ratio are associated with higher risk of stroke [15]. However, most published systematic reviews and meta-analyses on the relationship between dietary sodium, potassium, and CVD are done among adults regardless of age, ignoring the complexity of older people. Thus, this review aims to examine the relationship of sodium and potassium intake and CVD in older people (>60 years).

## 2. Materials and Methods 

The present systematic review was reported according to the Preferred Reporting Items for Systematic Reviews and Meta-Analyses (PRISMA) statement [19].

### 2.1. Search Strategy

PubMed and Web of Science were systematically searched from January 2015 up to 10 July 2020, to assess the most recent evidence. The search was performed by one author (CG) using a combination of MeSH terms and keywords related to population, CVD, and dietary sodium and potassium intake, with no restriction on language. The search terms used in PubMed was the following, and then adapted to Web of Science: (“sodium” OR “sodium chloride”) AND (“potassium”) AND (“cardiovascular disease” OR “coronary heart disease” OR “ischemic heart disease” OR “myocardial infarction” OR “stroke” OR “heart attack” OR “hypertension” OR “high blood pressure”) AND (“elderly” OR “older adults” OR aging OR “later life” OR senior OR nonagenarian OR octogenarian OR centenarian). The reference lists of included articles and reviews were also manually reviewed for additional relevant studies.

### 2.2. Eligibility Criteria and Study Selection

Studies were included in this review if: (1) the study was published in English, French, Portuguese, or Spanish; (2) the study design was cohort, case-cohort, nested case-control, case-control with a follow up of 12 months’ length or above, cross-sectional, or intervention studies with randomized groups (the intervention/experimental group and the control group with no intervention/usual sodium or potassium intake), and a minimum intervention duration of 4 weeks; (3) participants’ mean or median age were ≥ 60 years old, living in the community or institutionalized (nursing home) (for longitudinal studies mean age were considered at baseline; studies with stratified analyses by age groups were considered if results were available for ages ≥ 60 years); (4) the exposure of interest was sodium, potassium, or both assessed through dietary questionnaires or urinary measurements (for intervention studies the intervention was performed using potassium-containing supplements or through dietary modification only with sodium or potassium as target components); (5) the outcome of interest was CVD (prevalence, incidence, or mortality), hypertension, systolic blood pressure (SBP), or diastolic blood pressure (DBP). We excluded studies performed among participants with kidney disease and studies that not exclusively targeted potassium or sodium intake but multiple health behaviors (e.g., physical activity and diet). If multiple studies were published based on the same sample, we chose to include the study reporting prospective analysis or the study that used the largest sample size (for cross-sectional analyses). The review of titles and abstracts of all identified studies, and full-text assessment were done independently by the authors (CG and SA). Any disagreement was resolved through consensus.

### 2.3. Data Extraction

Data extraction of eligible studies was done independently by both authors using a standardized form, with any disagreement resolved by consensus. The information extracted comprised: the first author‘s last name, publication year, country, study design, mean or median age of participants, number of participants/case, sex, exposure identification and assessment method, outcome measure identification, follow-up duration, covariates used in multivariable analysis, and results. For intervention studies, details about the intervention was also extracted.

### 2.4. Quality Assessment

Both authors independently assessed the risk of bias for observational studies using the Risk of Bias Assessment Tool for Nonrandomized Studies (RoBANS) [20], and the revised Cochrane risk-of-bias tool (RoB 2 tool) for randomized trials [21]. RoBANS evaluated six bias domains: (1) selection of participants; (2) confounding variables; (3) measurement of exposure; (4) outcome assessments; (5) incomplete outcome data; and (6) selective outcome reporting. The risk of bias for each domain was judged as low risk, high risk, or unclear risk. Overall risk of bias was evaluated according to three key domains (the selection of participants, confounding variables, and incomplete outcome data). Then, if one of the three domains was assessed having a risk of bias as low, unclear, or high overall risk was classified according the more frequent classification. If each key domain was assessed differently the overall risk was categorized as unclear.

RoB tool considered the assessment of bias in five domains due to (1) randomization process, (2) deviations from intended interventions, (3) missing outcome data, (4) measurement of the outcome, and (5) selection of the reported results. The possible risk-of-bias judgements in each domain are low risk of bias, some concerns or high risk of bias. Additionally, an overall risk-of-bias judgement was done according to each domain assignment. Therefore high risk of bias was assigned if study it was judged to be at high risk of bias for at least one domain, some concerns if the study it was judged to raise some concerns for at least one domain and low risk if the study it was judged to be at low risk of bias for all domains.

## 3. Results

**Figure 1:**
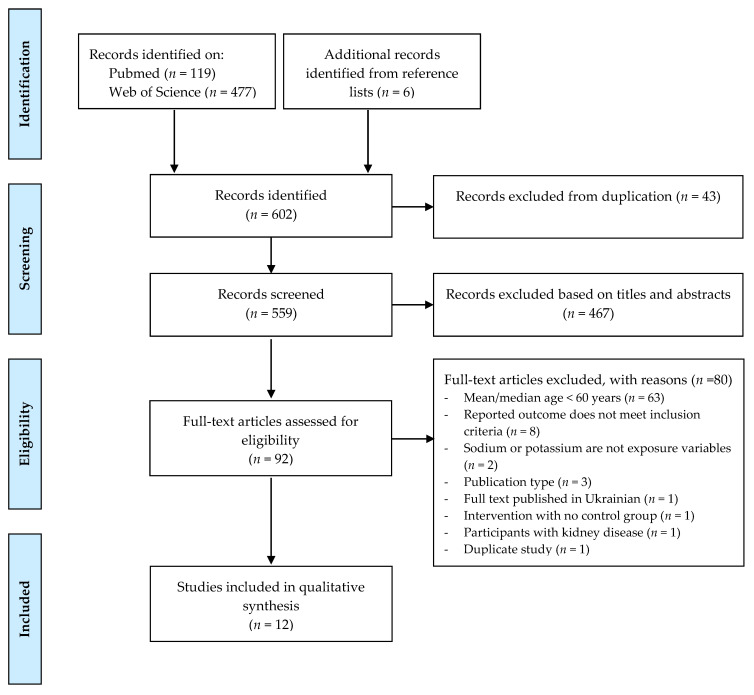
Flowchart of systematic literature search for inclusion in the review of relationship of sodium and potassium intake and cardiovascular disease among older people.

The database search resulted in a total of 596 records. Additionally, six papers were identified from the reference list. After the removal of duplicates (*n* = 43), the titles and abstracts of 559 records were screened according to eligibility criteria. Then, from these, the full-text of 92 publication were screened, and 12 papers were included in the present review for data extraction and quality assessment (Figure 1).

### 3.1. Characteristics of Studies

Table 1, Table 2 and Table 3 show the characteristics of the 12 included studies, ordered on publication year, which examined the association between sodium and/or potassium intake and CVD among older people. All included studies were carried out among population from developed countries, including USA (*n* = 5), France (*n* = 1), Italy (*n* = 1), Japan (*n* = 1), Korea (*n* = 1), the Netherlands (*n* = 1), Poland (*n* = 1), and Turkey (*n* = 1). Participants mean age ranged 60 to 79.7 years and all studies were conducted in men and women. Some studies were restricted to population with a specific condition, including type 2 diabetes (*n =* 1) [22], acute stroke (*n* = 1) [23], hypertension (*n* = 1) [24], and pre-hypertension (*n* = 1) [25].

From the 12 included studies, the majority are prospective studies (*n* = 6) [22,26,27,28,29,30] and cross-sectional studies (*n* = 5) [23,24,31,32,33], and only one was an experimental study [25]. Most of the studies had as exposure variable sodium-to-potassium ratio (*n* = 5) [23,27,28,30,33] and both potassium and sodium intake (*n* = 5) [22,23,25,31,33]. Of the 11 observational studies included, the exposure variable was assessed by self-reported questionnaires (*n* = 5) [24,26,27,28,31], spot urine (*n* = 4) [22,23,30,32], and 24-h urinary excretion (*n* = 2) [29,33]. Regarding outcome, most of the studies analyzed cardiovascular events (*n* = 9) [22,23,26,27,28,29,30,31], three BP [25,32], and one both [24,33]. Outcome assessment were derived from medical records [22,26,27,29,31], death certificates [13,22,28], BP monitors [24,25,28,32,33], and self-reported data [24,30]. One study not reported how outcome variable (i.e., stroke) was assessed [23].

### 3.2. Quality Assessment

**Figure 2:**
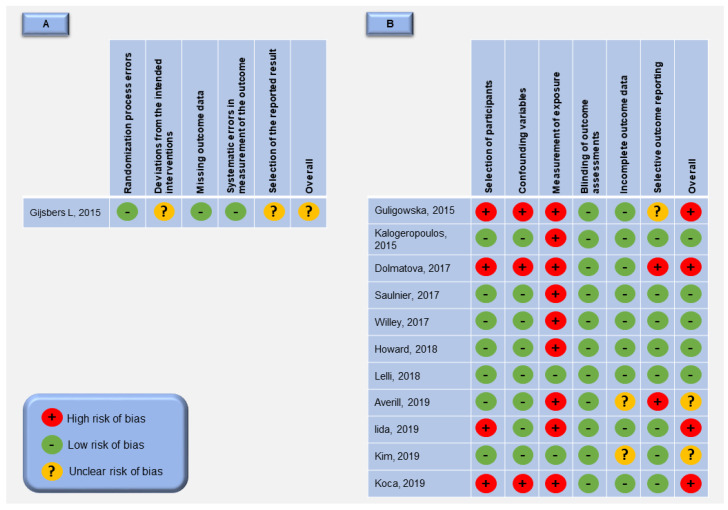
Risk of bias summary: review authors’ judgements about each risk of bias item for each included study. (**A**)—randomized controlled trials included; (**B**)—non-randomized studies included.

For the 12 selected studies, the overall risk of bias was rated high for four studies [23,24,31,32], unclear for three studies [20,25,30] and low for five studies [22,26,27,28,29] (Figure 2). The randomized controlled trial included [25] has low risk of bias in the “randomization process errors”, “missing outcome data” and “systematic errors in measurement of the outcome” domains of the quality assessment. However, the “deviations from the intended interventions” domain was rated unclear because this is a cross-over trial and does not include a washout period between the treatments as a mean of reducing the carry-over effect. The “selection of the reported result” domain was also rated unclear mainly due to the impossibility to access the baseline differences for all outcome variables in the randomized groups at the start of the cross-over trial. For the 11 observational studies included, the “selection of participants” was judged as low risk of bias, except for four studies due to retrospective data collection and unclear disease diagnosis assessment [31], generation of patients definition by self-report [24], unclear confirmation of excluded patients from control group [32] and insufficient data to prove that case and control group are from a comparable population group [23]. The “confounding variables” domain was only judged as high risk of bias in three studies [23,24,31], mainly due to the fact that major confounding variables (age, gender, body mass index, smoking, alcohol consumption, and race/ethnic group) were not considered. In relation to “incomplete outcome data” domain, two studies was judged unclear risk of bias [20,30], because it is uncertain whether the incomplete outcome data could affect the study outcome or not present data about differences between included and excluded subjects. Finally, the “measurement of exposure” domain was considered as low risk if sodium and/or potassium intake was estimated by 24-hour urinary collection considered the “gold standard” method [20,29], other studies estimates these variables by spot urines, dietary recall or food records and these methods may underestimate sodium consumption and/or do not consider the salt used in food preparations [34,35,36,37].

### 3.3. Sodium Intake 

A total of nine studies analyzed sodium intake, which five are cross-sectional studies [23,24,31,32,33], three are prospective studies [22,26,29] and one is an experimental study [25]. Kyung Kim et al. [33] evaluated the dose-response association of 24-h urine sodium excretion and 24-h ambulatory blood pressure among community-based participants and found that nighttime BP linearly increased with 24-h urine sodium; however, daytime BP and 24-hour systolic BP (SBP) showed a significant non-linear relationship with sodium excretion. Iida et al. [32] explored the association of salt intake (NaCl) with BP and showed that estimated salt intake (per g/day) (per one-unit mean or standard deviation-SD) was associated with higher SBP, but not with diastolic BP (DBP). In the other hand, in another cross-sectional study [24] in adjusted models no association were found between sodium consumption and cardiovascular events history and higher BP. Furthermore, cross-sectional studies of Guligowska et al. [31] and Koca et al. [23] found that sodium was lower in patients with myocardial infarction and stroke compared to their counterparts, respectively.

From prospective cohort studies, two reported no significant association with cardiovascular events as the result of higher sodium intake [26,29] and one reported increased risk of cardiovascular death as the result of lower urinary sodium concentration [22]. Saulnier et al. [22] evaluated the relationship between spot urine sodium concentration and mortality and cardiovascular death among type 2 diabetes patients and reported that an increase of one SD was associated with a decrease of 22% of cardiovascular death.

Gijsberg et al. [25] conducted a randomized, placebo-controlled crossover study of 36 untreated pre-hypertensive adults who were randomly assigned to three times in one of the following three groups: sodium supplementation (3 g/day, equals 7.6 g/day of salt), potassium supplementation (3 g/day) or placebo. Three times consecutive intervention periods of four weeks, without washout, were conducted for each of these groups. During sodium supplementation, office and 24 h-ambulatory SBP and DPB were significantly increased to ~8 mmHg and ~3 mmHg compared to placebo period, respectively.

### 3.4. Potassium Intake

Potassium intake was explored in three cross-sectional studies [23,31,33], two prospective studies [22,27] and one experimental study [25]. Among cross-sectional studies, two studies did not find significant results between potassium intake and cardiovascular events [31] or BP [33]. Koca et al. [23] showed that urinary potassium excretion was significantly lower in male patients with stroke compared to healthy counterparts. Willey et al. [27] explored data from the Northern Manhattan Study (NOMAS) and stratified results according to sodium intake. Among those with sodium intake <2300mg/d there was a positive association between potassium intake, total, and ischemic stroke, whereas among those with sodium intake ≥2300 mg/d a marginally inverse association was observed between potassium intake and stroke. Potassium intake was not the main outcome in the other prospective study [22]; however, lower tertiles of urinary potassium concentration was associated with higher cardiovascular mortality. In the experimental study, Gijsberg et al. [25] found during the potassium supplementation that 24-h ambulatory SBP and DBP were significantly reduced by ~4 mmHg and ~2 mmHg, respectively.

### 3.5. Sodium-to-Potassium Ratio

Most of the studies that explored sodium-to-potassium ratio are prospective studies [27,28,30] and two are cross-sectional studies [20,23]. All of the prospective studies reported increased risk of cardiovascular events as the result of higher sodium-to-potassium ratio. Data from Multi-Ethnic Study of Atherosclerosis (MESA) conducted among 6814 adults from different ethnic groups found in full-adjusted model that a sodium-to-potassium ratio higher than one was associated with the risk of stroke [30]. Likewise, Wiley et al. [27] reported a higher sodium-to-potassium ratio was associated with a greater risk of ischemic and total stroke. Howard et al. [28] conducted a longitudinal cohort study among adults’ aged 45 years or older and explored results according to sex and black and white participants. Among men and women, the sodium to potassium ratio was associated with incident hypertension. Additionally, among black men and women the ratio of sodium to potassium accounted for 12.3% and 6.8% of the excess risk of hypertension, respectively. Kyung Kim et al. [33] also found a cross-sectional linear association between nighttime BP and 24-hour BP with sodium-to-potassium ratio. Urinary sodium-to-potassium ratio was not significantly different between stroke and control groups in Koca et al.’s study [23].

## 4. Discussion

The present review provides summarized evidence on the association between sodium and potassium intake with CVD and hypertension among older people. We identified five non-randomized trials with low bias risk and one randomized controlled trial with uncertain risk of bias that assessed the impact of sodium, potassium, and sodium-to-potassium ratio on CVD. We found inconsistent results that supports the recommendation to reduce sodium intake in this population, however we found strong evidence to support the increase of potassium intake and the decrease of sodium-to-potassium ratio in reducing the risk of hypertension and CVD, particularly stroke.

In the analysis of sodium intake, our findings shown a lack of evidence of an effect of dietary sodium reduction on cardiovascular events and mortality that is in accordance with other authors [38]. Two included studies with low risk of bias shows that dietary sodium intake was not associated with incident of CVD or heart failure [26,29], one study indicate that low sodium intake increased the risk of CVD and mortality [22] and the other related increased BP with increasing sodium intake [25]. This non-linear association between sodium excretion and mortality was already described, suggesting a J-shaped association between sodium intake and cardiovascular events recommending a moderate sodium intake in the general population (3–5 g/d), with targeting the lower end of the moderate range among those with hypertension [39]. Saulnier et al. [22] found that low urinary sodium was associated with an increased risk of CVD and mortality in type 2 diabetes older persons and Lelli et al. [29] found similar association with mortality but not with CVD in frail participants. Diabetes and frailty are two prevalent conditions in older persons, and both have a recognized influence on studied outcomes. The low sodium excretion maybe due to insulin therapy, because the antinatriuretic effect of insulin may contribute to the relationship between insulin resistance and hypertension, the authors also raises the question if kidneys ability to excrete sodium and urine is implicated in the occurrence of fatal cardiovascular diseases [22]. The activation of sympathetic nervous system and the renin–angiotensin–aldosterone system, implicated in the regulation of sodium and volemia, are stimulated in frail persons, which may lead to increased risk for cardiovascular events [29,40]. Another mechanism that could explain the association of the risk of mortality and low sodium intake in frail persons is the lower caloric intake and malnutrition compromising cellular metabolism and several biological processes [41]. Discrepant findings in studies can be also attributable to differences in ranges of sodium intake, study populations and methods of sodium assessments. Some of the included studies uses food frequency questionnaire to estimates sodium intake leading to an underestimation of sodium intake and of an attenuated association between sodium and the outcome [36]. Furthermore, another study used one single spot urine without consideration to the circadian pattern of sodium excretion [42]. Although spot urine and dietary questionnaires may provide useful population sodium estimates, they are poor predictors at individual level [36]. Finally, older adults are inherently at higher risk for CVD and mean sodium intake are lower, mainly due to lower caloric intake; therefore; the effect of high sodium might have been more difficult to ascertain [43]. Although our inconsistent results for the dietary sodium effect on CVD among older people, a large body of evidence showed a favorable effect of low salt consumption on CVD, organ damage, and blood pressure and support the public health recommendation that adult population likely benefit from reducing sodium intake [44,45,46,47].

Regarding potassium intake analysis, increase potassium intake seems to be protective to CVD, likewise other meta-analyses verified similar findings [48,49]. Moreover, those persons with high sodium consumption could achieve great benefit with increased intake of potassium-rich foods to lower stroke risk [27,50]. In the other hand, the relationship between dietary potassium and BP seems to be not linear but U-shaped with stronger lowering-BP effect among those with high sodium intake as reported in a recent meta-analysis of randomized clinical trial [15], providing important insight about the combined effect of sodium and potassium intake. The combined association of sodium and potassium was explored as sodium-to-potassium ratio in five of the 12 included studies suggesting an increased risk of CVD and BP with higher sodium-to-potassium ratio. Likewise, it has been suggested that the sodium-to-potassium ratio is more strongly associated with BP than either sodium or potassium alone, being considered a superior metric in the evaluation of BP and incident hypertension [51]. Sodium and potassium are significant regulators of BP. Dependent of pre-exiting electrolyte balance, particularly sodium, reduced potassium intake leads to sodium retention, down regulation of vascular sensitivity to catecholamines, stimulated renin activity and worsening endothelial function [52].

Such as previous systematic reviews of dietary interventions, we observed marked heterogeneity across studies in terms of their population, sample size, methods, and follow-up. Regarding observational studies, few studies included covariates as controlled or well-characterized diet (influences of alcohol, coffee, salt addition in cooking, and DASH scores), dietary supplementation, or physical activity characterization that may influence the association between dietary sodium, potassium, and CVD. Additionally, other potentially important confounders or effect modifiers mediating factors [28] were not taken in consideration such as gender, education level/socioeconomic status, body mass index, waist circumference or stress. Race was also considered only in few studies [26,27,28,30], all from USA.

Our review has several strengths, including the most up-to-date synthesis of evidence on this topic and focus on older persons. We conducted the review according to guidance from the PRISMA statement. The main limitation of this review is that relevant studies may have been missed since we used only two databases for literature search. However, the PubMed and Web of Science have been used broadly as primary databases in review on nutrition area. Additionally, searching in multiple databases may cover more primary studies than using a single database [53]. Moreover, the inclusion of observational studies do not allow drawing any conclusions related to cause and effect.

Our findings suggests that salt reduction advice in older persons should take in consideration the previous characterization of usual level of consumption, for example by sodium excretion in 24-h urinary samples, in order to recommends reduction to ones that ingest more than 2000 mg/d. This population could benefit with increased potassium-rich foods in daily diet in order to optimize sodium-to-potassium ratio. Frailty, diabetes, ethnicity, and literacy could have an important role in the mediation of these outcomes.

## 5. Conclusions

The present review suggests that both higher potassium and lower sodium-to-potassium ratio are associated with lower risk of CVD. In contrast, our findings show a lack of evidence of dietary sodium reduction affecting CVD, however, considering the quality of the included studies these results should be interpreted with caution. To strengthen the study of these relationships further rigorous, large long-term, and randomized controlled trials are necessary.

## Figures and Tables

**Table 1:** Characteristics of included cross-sectional studies in systematic review of dietary sodium, potassium, sodium-to-potassium ratio, and cardiovascular disease.

Author, Year [Reference]	Country	Participants Characteristics	Exposure	Sodium/Potassium Intake Assessment	Outcome Measures	Covariates	Main Findings
Guligowska AR, 2015 [31]	Poland	*n* = 239 (66 men) mean age = 72.0 ± 9.34 years	Sodium Potassium	24-hour recall questionnaire	Cardiometabolic disease (hypertension, history of ischemic heart disease, chronic HF or MI)	None	No significant differences for dietary sodium and potassium were found between participants with hypertension or disease history and healthy peers, except for sodium intake that was lower in patients with a history of MI (2680 ± 1019 mg vs. 3471 ± 1242 mg, *p* = 0.010) compared to their counterparts.
Dolmatova EV, 2018 [24]	USA	*n* = 13,033 with self -reported hypertension (6910 men) mean age = 60 ± 14 years	Sodium	24-h recall questionnaire	History of MI, HF, stroke BP	Age	In univariate analysis lower sodium consumption was found among adults with a history of MI, HF, and stroke (*p* < 0.001) but the difference did not remain significant after adjustment for age. Higher SBP and lower DBP were associated with higher sodium in univariate analysis, but the difference was no longer significant after adjustment for age.
Iida, 2019 [32]	Japan	*n* = 288 (116 men) mean age = 79.7 ± 4.2 years	Salt (NaCl)	Spot urine samples	BP	Age, sex, height, body weight, smoking status, PA, comorbidity (cardiovascular, cerebrovascular, and renal diseases), diabetes mellitus, dyslipidemia, alcohol intake, and medication (antihypertensive agents and diuretics)	A one-unit higher value in estimated salt intake (per g/d) was associated with a higher SBP (adjusted difference: 1.73 mmHg, 95% CI 0.71 to 2.76 mmHg). One SD higher value in estimated salt intake (per g/d) was also associated with a higher SBP (adjusted difference: 4.13 mmHg, 95% CI 1.69 to 6.57 mmHg). A one-unit or SD higher values in estimated salt intake (per g/d) were not associated with higher DPB.
Kyung Kim, 2019 [33]	Korea	*n* = 217 (94 men) median age = 60 (IQR: 57-63)	Sodium Potassium Sodium to potassium ratio	24-hour urine excretion	24-hour ambulatory BP	Age, gender, BMI, smoking, and use of antihypertensive medications	Nighttime blood pressure linearly increased with 24-h urine sodium (SBP: β = 0.1706, 95% CI 0.0361–0.3052; DBP: β = 0.1440, 95% CI 0.0117–0.2763) and the sodium to potassium ratio (SBP: β = 0.1415, 95% CI 0.0127–0.2703; DBP: β = 0.1441 95% CI 0.0181–0.2700). The 24-h BP was linearly increased with sodium to potassium ratio (SBP: β = 0.1325, 95% CI 0.0031–0.2620; DBP: β = 0.1234 95% CI 0.0025–0.2444). Non-linear associations were found between daytime blood pressure (SBP and DBP), 24-hour SBP and sodium (*p* < 0.05).
Koca TT, 2019 [23]	Turkey	*n* = 82 (50 patients with stroke (28 men) and 32 controls (13 men)) mean age _stroke group_ = 65.9 ± 14.6 years mean age _contro groupl_ = 60.9 ± 14.1 years	Sodium Potassium Sodium-to-potassium ratio	Spot urine samples	Stroke	None	Urinary sodium to potassium ratio was not significantly different between stroke and control groups. Urinary potassium, sodium, and sodium to potassium ratio excretion was significantly lower in male patients with stroke compared to healthy male (*p* < 0.05 for all).

BP, blood pressure; BMI, body mass index; CI, confidence interval; DBP, diastolic blood pressure; HF, heart failure; MI, myocardial infarction; PA, physical activity; SD, standard deviation; SBP, systolic blood pressure; USA, United States of America; IQR, interquartile range.

**Table 2:** Characteristics of included longitudinal studies in systematic review of dietary sodium, potassium, sodium-to-potassium ratio, and cardiovascular disease.

Author, Year	Country	Participants Characteristics	Study Design	Follow-Up (Years)	Exposure	Sodium/Potassium Intake Assessment	Outcome Measures	Covariates	Main Findings
Kalogeropoulos AP, 2015 [26]	USA	*n* = 2642 (1290 men)	Prospective cohort	10	Sodium (as continuous variable and categorical variable into 3 groups: <1500 mg/d; 1500–2300 mg/d; >2300 mg/d)	Food frequency questionnaire (at the year 2 visit)	Incident CVD (n_cases_ = 572) (i.e., coronary heart disease (MI, angina, or coronary revascularization), cerebrovascular disease (stroke, transient ischemic attack, or symptomatic carotid artery disease), peripheral arterial disease	Age, sex, race, baseline hypertensive status, BMI, smoking status, PA, prevalent CVD (for HF events), pulmonary disease, diabetes mellitus, depression, BP, heart rate, electrocardiogram abnormalities, and serum glucose, albumin, creatinine, and cholesterol levels	Ten-year incident CVD, or incident HF, were not associated with sodium intake.
		mean age = 73.6 ± 2.9 years					Incident HF (n_cases_ = 398)		
Saulnier PJ, 2017 [22]	France	*n* = 1439 types 2 diabetes patients (835 men)	Prospective cohort	Median = 5.7 (IQR: 3.1–8.8)	Sodium (as continuous variable and categorical variable into tertiles: low, <69 mmol/L; intermediate, 69–103 mmol/L; high, >103 mmol/L) Potassium (as categorical variable into tertiles—not specified)	Spot urinary sample	Cardiovascular death (n_cases_ = 268)	Age, sex, urinary sodium and potassium, urine to plasma creatine ratio, estimated 24 h sodium excretion, BMI, history of urinary albumin to creatine concentration ratio, N-terminal pro-brain natriuretic peptide	It was found significant relationships between cardiovascular mortality, and sodium and potassium tertiles (Log-rank *p* < 0.001), with patients in the lower tertiles having the highest mortality. For each 1-SD increase of urinary sodium concentration in the adjusted model, cardiovascular mortality was 24% lower (HR: 0.76, 95% CI: 0.66–0.88).
		mean age = 65.3 ± 10.7 years							
Willey J, 2017 [27]	USA	*n* = 2496 (902 men)	Prospective cohort	Mean = 12 ± 5	Sodium to potassium ratio	Food frequency questionnaire (at baseline)	Incident stroke (n_cases_ = 268)	Age, sex, high-school completion, race ethnicity, total calories, Mediterranean diet score, moderate alcohol use, moderate heavy physical activity, smoking, estimated glomerular filtration rate, body mass index, hypertension, hypercholesterolemia, diabetes mellitus, sodium consumption	In adjusted models, a higher sodium:potassium ratio was associated with increased risk for stroke (HR: 1.6, 95% CI: 1.19–2.14) and ischemic stroke (HR: 1.58, 95% CI: 1.20–2.06).
		mean age= 68.7 ± 10 years (55% Hispanic)					Incident ischemic stroke (n_cases_ = 227)		Marginally positive association was observed for potassium intake and stroke among those with <2300 mg sodium/d and an inverse association was observed for potassium intake among those with ≥2300 mg sodium/d.
					Potassium (as continuous variables and quartiles)				
Lelli D, 2018 [29]	Italy	*n* = 920 (415 men)	Prospective cohort	9	Sodium	24-hour urinary excretion	Incident cardiovascular events (n_cases_ = 169)	Age, sex, education, estimated creatinine clearance, SBP, cigarette smoking, hypertension, diabetes, BMI, caloric intake/body weight, antihypertensive drugs, and diuretics	An association was found between 24-hour sodium excretion and cardiovascular disease (RR 0.95; 95% CI 0.90–1), which did not remain after adjustment for confounders (RR: 0.96, 95% CI: 0.90–1.02).
		mean age = 74.5 ± 6.99 years					(i.e., angina pectoris, myocardial infarction, heart failure, and stroke)		
Howard G, 2018 [28]	USA	*n* = 6897 (3125 men; 1807 black participants)	Prospective cohort	9.4	Sodium to potassium ratio	Food frequency questionnaire (at baseline)	Incident hypertension (n_cases_ = 836 (298 men) for black and 1679 (837 men) for white participants)	Age, race, and baseline systolic blood pressure for the risk factor of incident hypertension	Among men, the sodium to potassium ratio was associated with incident hypertension (OR: 1.11, 95% CI: 1.01 to 1.20; incidence proportion at 25th percentile, 32.9%, 95% CI: 30.4% to 35.5% and the 75th percentile, 35.8%, 95% CI: 33.5% to 38.2%; absolute risk difference between black and white participants, 2.9%, 95% CI: 0.4% to 5.5%). Among black men, the ratio of sodium to potassium accounted for 12.3% (95% CI: 1.1% to 22.8%) of the excess risk of hypertension.
		mean age = 62 ± 8 years							Among women, the sodium to potassium ratio was associated with incident hypertension (OR: 1.13, 95% CI: 1.04 to 1.22; incidence proportion at 25th percentile, 31.1%, 95% CI: 29.1% to 33.5% and the 75th percentile, 34.5%, 95% CI: 32.2% to 36.8%; absolute risk difference between black and white participants, 3.3%, 95% CI: 1.1% to 5.5%). Higher dietary ratio of sodium to potassium accounted for 6.8% (95% CI: 1.6% to 11.9%) of the risk of hypertension among black women.
Averill MM, 2019, USA [30]		*n* = 6705 (3160 men)	Prospective cohort	11.7 (±2.2)	Sodium to potassium ratio	Spot urine samples (at baseline)	Incident CVD (n_cases_ = 781) (MI, definite angina, stroke, transient ischemic attack, coronary heart disease death)	Age, sex, race, diabetes mellitus, smoking (current and former), total cholesterol, high-density lipoprotein cholesterol, treated hypertension, education, SBP, DBP, urine creatinine, hip circumference, BMI, aspirin use, intentional exercise, glomerular filtration rate, dietary energy intake, maximum of common carotid artery intimal medial thickness, and IL-6 (interleukin 6) levels	After adjustment, only sodium-to-potassium ratio >1 was associated with the risk of stroke (HR: 1.47, 95% CI: 1.07–2.00).
		mean age= 61.2 ± 10.2					Incident coronary heart disease (n_cases_ = 530) (MI and angina)		
							Incident HF (n_cases_ = 274)		
							Incident peripheral vascular disease (n_cases_ = 104)		
							Incident stroke (n_cases_ = 236)		
							SBP		

BP, blood pressure; BMI, body mass index; CI, confidence interval; CVD, cardiovascular disease; DBP, diastolic blood pressure; HF, heart failure; HR, Hazard ration; MI, myocardial infarction; PA, physical activity; SD, standard deviation; SBP, systolic blood pressure; USA, United States of America; IQR, interquartile range; OR, odds ratio; RR, relative risk.

**Table 3:** Characteristics of included trials in systematic review of dietary sodium, potassium, sodium-to-potassium ratio, and cardiovascular disease.

Author, Year	Country	Participants Characteristics	Follow-Up	Study Design	Intervention Details	Outcome Measures	Main Findings
Gijsbers L, 2015 [25]	The Netherlands	*n* = 36 untreated (pre)hypertensive (24 men) mean age = 65.8 years	4 weeks	Randomized, double-blind, placebo-controlled crossover	After a 1-week run-in period, subjects were randomly allocated to 3 times in one of the treatments: sodium supplementation (3 g/day, equals 7.6 g/day of salt), potassium supplementation (3 g/day) or placebo.	Fasting office BP 24-h ambulatory BP	During sodium supplementation, office and 24h-ambulatory were significantly increased to ~ 8 mmHg and ~4 mmHg, respectively. During potassium supplementation, 24-h ambulatory SBP and DBP was significantly reduced by ~4 mmHg and ~2 mmHg, respectively.

BP, blood pressure; DBP, diastolic blood pressure; SBP, systolic blood pressure.

## References

[1] United Nations (2017). World Population Ageing 2017: Highlights.

[2] Abrignani M.G., Aiello A., Colivicchi F., Lucà F., Fattirolli F., Gulizia M.M., Nardi F., Pino P.G., Gregorio G. (2020). Cardiovascular prevention in the elderly: Limitations and opportunities. G. Ital. Cardiol. (Rome).

[3] World Health Organization (2013). Global Action Plan for the Prevention and Control of Noncommunicable Diseases 2013–2020.

[4] Wilkins E., Wilson L., Wickramasinghe K., Bhatnagar P., Leal J., Luengo-Fernandez R., Burns R., Rayner M., Townsend N. (2017). European Cardiovascular Disease Statistics 2017.

[5] Mendis S., Puska P., Norrving B., World Health Organization (2011). Global Atlas on Cardiovascular Disease Prevention and Control.

[6] Yazdanyar A., Newman A.B. (2009). The Burden of Cardiovascular Disease in the Elderly: Morbidity, Mortality, and Costs. Clin. Geriatr. Med..

[7] Damluji A.A., Ramireddy A., Otalvaro L., Forman D.E. (2015). Secondary cardiovascular prevention in older adults: An evidence based review. J. Geriatr. Cardiol..

[8] Benetos A., Petrovic M., Strandberg T. (2019). Hypertension Management in Older and Frail Older Patients. Circ. Res..

[9] Schall P., Wehling M. (2011). Treatment of arterial hypertension in the very elderly: A meta-analysis of clinical trials. Arzneimittel-Forschung.

[10] Whelton P.K., Carey R.M., Aronow W.S., Casey D.E., Collins K.J., Himmelfarb C.D., DePalma S.M., Gidding S., Jamerson K.A., Jones D.W. (2018). 2017 ACC/AHA/AAPA/ABC/ACPM/AGS/APhA/ASH/ASPC/NMA/PCNA guideline for the prevention, detection, evaluation, and management of high blood pressure in adults: A report of the American College of Cardiology/American Heart Association Task Force on Clinical Practice Guidelines. J. Am. Coll. Cardiol..

[11] Williams B., Mancia G., Spiering W., Agabiti Rosei E., Azizi M., Burnier M., Clement D.L., Coca A., de Simone G., Dominiczak A. (2018). 2018 ESC/ESH Guidelines for the management of arterial hypertension: The Task Force for the management of arterial hypertension of the European Society of Cardiology (ESC) and the European Society of Hypertension (ESH). Eur. Heart J..

[12] Aune D., Giovannucci E., Boffetta P., Fadnes L.T., Keum N., Norat T., Greenwood D.C., Riboli E., Vatten L.J., Tonstad S. (2017). Fruit and vegetable intake and the risk of cardiovascular disease, total cancer and all-cause mortality—A systematic review and dose-response meta-analysis of prospective studies. Int. J. Epidemiol..

[13] He F.J., MacGregor G.A. (2010). Reducing Population Salt Intake Worldwide: From Evidence to Implementation. Prog. Cardiovasc. Dis..

[14] O’Donnell M., Mente A., Rangarajan S., McQueen M.J., O’Leary N., Yin L., Liu X., Swaminathan S., Khatib R., Rosengren A. (2019). Joint association of urinary sodium and potassium excretion with cardiovascular events and mortality: Prospective cohort study. BMJ.

[15] Jayedi A., Ghomashi F., Zargar M.S., Shab-Bidar S. (2019). Dietary sodium, sodium-to-potassium ratio, and risk of stroke: A systematic review and nonlinear dose-response meta-analysis. Clin. Nutr..

[16] Filippini T., Naska A., Kasdagli M., Torres D., Lopes C., Carvalho C., Moreira P., Malavolti M., Orsini N., Whelton P.K. (2020). Potassium Intake and Blood Pressure: A Dose-Response Meta-Analysis of Randomized Controlled Trials. J. Am. Hear. Assoc..

[17] World Health Organization (2012). Guideline: Sodium Intake for Adults and Children.

[18] World Health Organization (2012). Guideline: Potassium Intake for Adults and Children.

[19] Moher D., Liberati A., Tetzlaff J., Altman D.G., Group P. (2009). Preferred reporting items for systematic reviews and meta-analyses: The PRISMA statement. PLoS Med..

[20] Kim S.Y., Park J.E., Lee Y.J., Seo H.J., Sheen S.S., Hahn S., Jang B.H., Son H.J. (2013). Testing a tool for assessing the risk of bias for nonrandomized studies showed moderate reliability and promising validity. J. Clin. Epidemiol..

[21] Sterne J.A.C., Savović J., Page M.J., Elbers R.G., Blencowe N.S., Boutron I., Cates C.J., Cheng H.-Y., Corbett M.S., Eldridge S.M. (2019). RoB 2: A revised tool for assessing risk of bias in randomised trials. BMJ.

[22] Saulnier P.-J., Gand E., Ragot S., Bankir L., Piguel X., Fumeron F., Rigalleau V., Halimi J.-M., Marechaud R., Roussel R. (2017). Urinary Sodium Concentration Is an Independent Predictor of All-Cause and Cardiovascular Mortality in a Type 2 Diabetes Cohort Population. J. Diabetes Res..

[23] Koca T.T., Tugan C.B., Seyithanoglu M., Koçyiğit B.F. (2019). The Clinical Importance of the Plasma Atherogenic Index, Other Lipid Indexes, and Urinary Sodium and Potassium Excretion in Patients with Stroke. Eurasian J. Med..

[24] Dolmatova E.V., Moazzami K., Bansilal S. (2018). Dietary sodium intake among US adults with hypertension, 1999–2012. J. Hypertens..

[25] Gijsbers L., Dower J.I., Mensink M., Siebelink E., Bakker S.J.L., Geleijnse J.M. (2015). Effects of sodium and potassium supplementation on blood pressure and arterial stiffness: A fully controlled dietary intervention study. J. Hum. Hypertens..

[26] Kalogeropoulos A.P., Georgiopoulou V.V., Murphy R.A., Newman A.B., Bauer D.C., Harris T.B., Yang Z., Applegate W.B., Kritchevsky S.B. (2015). Dietary sodium content, mortality, and risk for cardiovascular events in older adults: The Health, Aging, and Body Composition (Health ABC) Study. JAMA Intern. Med..

[27] Willey J., Gardener H., Cespedes S., Cheung Y.K., Sacco R.L., Elkind M.S. (2017). Dietary Sodium to Potassium Ratio and Risk of Stroke in a Multiethnic Urban Population: The Northern Manhattan Study. Stroke.

[28] Howard G., Cushman M., Moy C.S., Oparil S., Muntner P., Lackland D.T., Manly J.J., Flaherty M.L., Judd S.E., Wadley V.G. (2018). Association of Clinical and Social Factors With Excess Hypertension Risk in Black Compared With White US Adults. JAMA.

[29] Lelli D., Antonelli-Incalzi R., Bandinelli S., Ferrucci L., Pedone C. (2018). Association between Sodium Excretion and Cardiovascular Disease and Mortality in the Elderly: A Cohort Study. J. Am. Med. Dir. Assoc..

[30] Averill M.M., Young R.L., Frazier-Wood A.C., Kurlak E.O., Kramer H., Steffen L.M., McClelland R.L., Delaney J.A., Drewnowski A. (2019). Spot Urine Sodium-to-Potassium Ratio Is a Predictor of Stroke. Stroke.

[31] Guligowska A., Pigłowska M., Śmigielski J., Kostka T. (2015). Inappropriate pattern of nutrient consumption and coexistent cardiometabolic disorders in elderly people from Poland. Pol. Arch. Intern. Med..

[32] Iida H., Kurita N., Takahashi S., Sasaki S., Nishiwaki H., Omae K., Yajima N., Fukuma S., Hasegawa T., Fukuhara S. (2019). Salt intake and body weight correlate with higher blood pressure in the very elderly population: The Sukagawa study. J. Clin. Hypertens.

[33] Kim M.K., Kwon M., Rhee M.-Y., Kim K.-I., Nah D.-Y., Kim S.-W., Gu N., Sung K.-C., Hong K.-S., Cho E.-J. (2019). Dose–response association of 24-hour urine sodium and sodium to potassium ratio with nighttime blood pressure at older ages. Eur. J. Prev. Cardiol..

[34] Mattes R.D., Donnelly D. (1991). Relative contributions of dietary sodium sources. J. Am. Coll. Nutr..

[35] Gonçalves C., Abreu S., Padrão P., Pinho O., Graça P., Breda J.J., Santos R., Moreira P. (2016). Sodium and potassium urinary excretion and dietary intake: A cross-sectional analysis in adolescents. Food Nutr. Res..

[36] McLean R.M. (2014). Measuring Population Sodium Intake: A Review of Methods. Nutrients.

[37] He F.J., Campbell N.R.C., Ma Y., MacGregor G.A., Cogswell M.E., Cook N.R. (2018). Errors in estimating usual sodium intake by the Kawasaki formula alter its relationship with mortality: Implications for public health. Int. J. Epidemiol..

[38] Taylor R.S., Ashton K.E., Moxham T., Hooper L., Ebrahim S. (2011). Reduced dietary salt for the prevention of cardiovascular disease. Cochrane Database Syst. Rev..

[39] O’Donnell M., Mente A., Yusuf S. (2015). Sodium Intake and Cardiovascular Health. Circ. Res..

[40] Verma S., Gupta M., Holmes D.T., Xu L., Teoh H., Gupta S., Yusuf S., Lonn E.M. (2011). Plasma renin activity predicts cardiovascular mortality in the Heart Outcomes Prevention Evaluation (HOPE) study. Eur. Hear. J..

[41] McCullough P.A., Fallahzadeh M.K., Hegazi R.M. (2016). Nutritional Deficiencies and Sarcopenia in Heart Failure: A Therapeutic Opportunity to Reduce Hospitalization and Death. Rev. Cardiovasc. Med..

[42] Johnston J.G., Pollock D.M. (2018). Circadian regulation of renal function. Free. Radic. Biol. Med..

[43] Moreira P., Sousa A.S., Guerra R.S., Santos A., Borges N., Afonso C., Amaral T.F., Padrão P. (2018). Sodium and potassium urinary excretion and their ratio in the elderly: Results from the Nutrition UP 65 study. Food Nutr. Res..

[44] Aburto N.J., Ziolkovska A., Hooper L., Elliott P., Cappuccio F.P., Meerpohl J.J. (2013). Effect of lower sodium intake on health: Systematic review and meta-analyses. BMJ.

[45] D’Elia L., La Fata E., Giaquinto A., Strazzullo P., Galletti F. (2020). Effect of dietary salt restriction on central blood pressure: A systematic review and meta-analysis of the intervention studies. J. Clin. Hypertens..

[46] D’Elia L., Galletti F., La Fata E., Sabino P., Strazzullo P. (2018). Effect of dietary sodium restriction on arterial stiffness: Systematic review and meta-analysis of the randomized controlled trials. J. Hypertens..

[47] Strazzullo P., D’Elia L., Kandala N.-B., Cappuccio F.P. (2009). Salt intake, stroke, and cardiovascular disease: Meta-analysis of prospective studies. BMJ.

[48] D’Elia L., Iannotta C., Sabino P., Ippolito R. (2014). Potassium-rich diet and risk of stroke: Updated meta-analysis. Nutr. Metab. Cardiovasc. Dis..

[49] D’Elia L., Barba G., Cappuccio F.P., Strazzullo P. (2011). Potassium intake, stroke, and cardiovascular disease a meta-analysis of prospective studies. J. Am. Coll. Cardiol..

[50] Du S., Batis C., Wang H., Zhang B., Zhang J., Popkin B.M. (2013). Understanding the patterns and trends of sodium intake, potassium intake, and sodium to potassium ratio and their effect on hypertension in China. Am. J. Clin. Nutr..

[51] Perez V., Chang E.T. (2014). Sodium-to-potassium ratio and blood pressure, hypertension, and related factors. Adv. Nutr..

[52] Kanbay M., Bayram Y., Solak Y., Sanders P.W. (2013). Dietary potassium: A key mediator of the cardiovascular response to dietary sodium chloride. J. Am. Soc. Hypertens..

[53] Vassar M., Yerokhin V., Sinnett P.M., Weiher M., Muckelrath H., Carr B., Varney L., Cook G. (2017). Database selection in systematic reviews: An insight through clinical neurology. Heal. Inf. Libr. J..

